# Supplementary material for: A pilot study of the Earable device to measure facial muscle and eye movement tasks among healthy volunteers
Source: PLOS Digit Health. 2022 Jun 30;1(6):e0000061. doi: 10.1371/journal.pdig.0000061 (PMC9931353; doi:10.1371/journal.pdig.0000061)
Supplement: S1 Fig — Architecture diagram of the final CNN implemented for activity classification. A single channel spectrogram computed from the segmented waveform is input to the model at classification time. A probability distribution over each of the 16 activities is output. The activity associated with the highest output likelihood estimate is inferred. (DOCX) [file pdig.0000061.s004.docx]

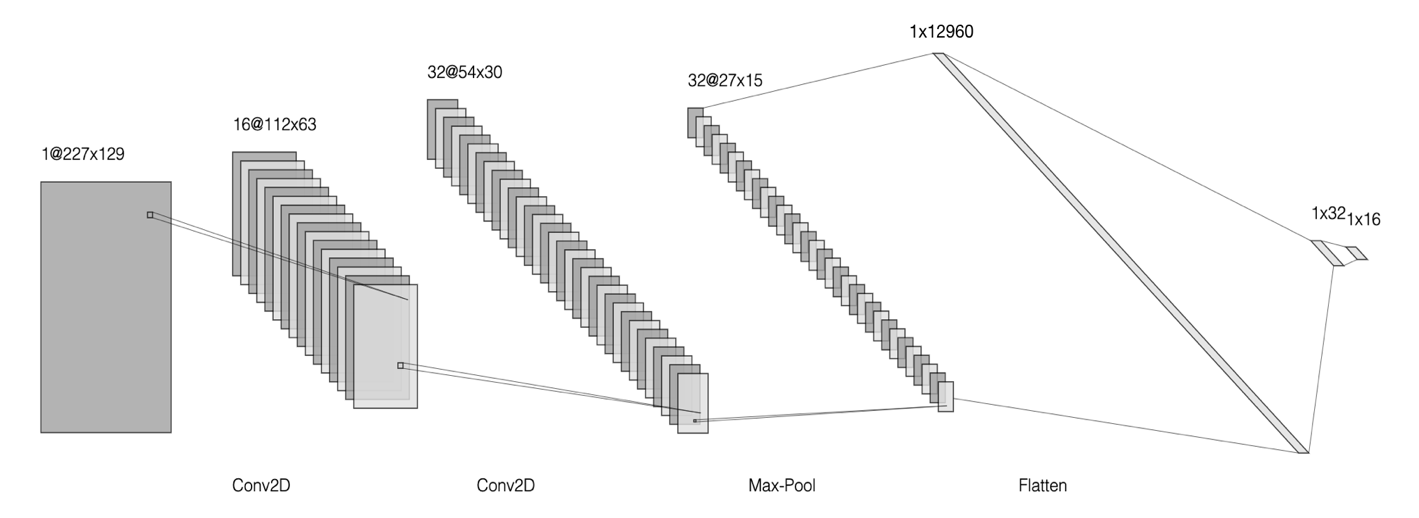


S1 Fig: Mock-PerfO Activity-Level CNN Classifier Architecture.

Architecture diagram of the final CNN implemented for activity classification. A single channel spectrogram computed from the segmented waveform is input to the model at classification time. A probability distribution over each of the 16 activities is output. The activity associated with the highest output likelihood estimate is inferred.
