# Supplementary material for: A pilot study of the Earable device to measure facial muscle and eye movement tasks among healthy volunteers
Source: PLOS Digit Health. 2022 Jun 30;1(6):e0000061. doi: 10.1371/journal.pdig.0000061 (PMC9931353; doi:10.1371/journal.pdig.0000061)
Supplement: Attachment — Submitted filename: Response to Reviewers.docx [file pdig.0000061.s005.docx]

**Response to Reviewers:**

**Reviewer #1:** This manuscript presents a pilot study that, according to the authors, determines whether the Earable device could be utilized to objectively measure facial muscle and eye movements. In particular, the paper investigates (1) whether raw EMG, EOG, and EEG Earable device signals could be processed to extract useful features in this context, (2) discuss Earable feature data quality, test re-test reliability, and statistical properties, (3) examine whether derived features could be used to determine the difference between various facial muscle and eye movement activities, and, (4) define what features and feature types are important for such activity level classification.

The manuscript is technically sound, and the presented data tend to support the manuscript conclusions. However, the pilot study only creates limited data, and in that context, it is not clear why the authors have decided to include classification approach that needs lots of data (e.g., CNN models), and compare it with classification approach that can cope with limited data (e.g., Random Forest) only to prove that former will provide better results.

**Author Response:** We thank the reviewer for this positive feedback regarding the presentation of results in this manuscript, and we are glad to hear that the conclusions drawn match the data and analysis methods presented. A major goal of this work is to discuss and present a framework for a wearable data analysis pipeline, and in this fit-for-purpose setting, it may be the case that a CNN does not make sense. At the onset of this work, this was not obvious to us. For example, while presenting this work at our institution, we have received questions about using the “raw data” as is several times from qualified parties (e.g., imaging and signal processing scientists). Further, it is not clear to us exactly how much data is enough for a CNN classification approach, vs a feature-based classification approach.

There are instances where wearable data collected in a clinical setting may make use of a CNN to answer a clinical question most appropriately. For example, Alias et al. deployed CNN architectures to classify gait activity of six subjects using surface EMG (sEMG) signals. (Alias, Needham et al. 2018) Each participant of this study performed only six trials of walking according to five different gait modes (walking at slow, normal, and fast speeds on a level surface and walking up and down an incline). Ultimately, their proposed CNN model achieved a gait classification accuracy of 77.95%, a 10.15% increase in accuracy as compared to their traditional, feature-based SVM model. Briouza et al. similarly proposed a shallow CNN architecture for high accuracy classification of hand movements when trained on sEMG data from the Ninapro-DB2 dataset (a popular hand-motion classification database consisting of sEMG signals of 49 hand motions from 40 participants) (Briouza, Gritli et al. 2021).

We believe that our strategy to analyze the raw data using a CNN model with the amount of data we have—representative of data that may be generated in a true clinical setting—is in fact a learning and therefore strength of this work. We have added the above justification to the methods to better help the reader understand our choice of including a CNN modeling approach, in addition to a feature-based classification approach.
**Reviewer #1:** In addition, due to small data sets, which is by the way point as limitation of the study, it would be good to present some calculations on internal consistency of obtained results prior to the discussion about possible generalization of the results.

**Author Response:** We thank the reviewer for highlighting the need to discuss internal consistency within the research study, given that it is run in healthy volunteers, and we want to make some (limited) generalizable claims at least about the data, for disease populations. A strength of this work is the study design, since it allows us to directly assess internal consistency. Internal consistency is a measure (typically a correlation) of how well repeats of the same “test” agree with each other when we have an expectation that they should agree.

In our study design (Supplementary Table 1), we have the same subject at the same time of day repeat each activity around 4 times. Our expectation is that these activity repeats (with the same subject, same activity, same time of day), should agree (be correlated) with each other. We use the Intraclass Correlation coefficient (ICC), with subjects as random effects, to assess the internal consistency of each task at each time of day. We have added the following sentence to the Results to clarify that ICC is a measure of internal consistency (lines 239 – 241): “Further, the ICC provides a measure of internal consistency for each activity, given our expectation that subjects should generate similar outputs for the same activity.”

**Reviewer #1:** The manuscript describes methodologically sound research with conclusions that are appropriately drawn based on the available data. However, it would be nice to further elaborate on the selection of initial set of activities (PerfOs) with a reference related to their importance for this kind of classification and activity recognition.

**Author Response:** We thank the reviewer for this important point. We have added the following to the Methods section (with relevant references) describing the rationale for the selection of the activities (PerfOs) used: Study tasks were based on activities performed during routine neurological examination of cranial nerves, as well as grading systems for facial weakness. These activities are commonly used to diagnose and assess severity of several diseases that affect cranial muscles, including facial nerve injury, stroke, and neuromuscular disorders such as myasthenia gravis and amyotrophic lateral sclerosis.

**Reviewer #1:** Further, the pilot study would benefit from integration of “non-healthy” subject(s) and their influence on the proposed quality of extracted features. Once again, this is already pointed out by the authors as limitation of the study.

**Author Response:** We of course agree that the inclusion of disease (non-healthy) subjects, as well as the enrollment of a larger cohort of control (healthy) subjects would be extremely beneficial. This would be beyond the scope of a pilot, though future work will absolutely focus on this, with the goal of using the analysis strategy presented in this work to derived disease signature. We highlight this under a paragraph in the discussion where we discuss limitations of this work, and describe what our next steps will be for future work.

**Reviewer #2:** This work studied the utility of earable devices in measuring facial muscle and eye movement tasks among healthy subjects and demonstrated that they can be used for classify mock-PerfO activities. The paper was written clearly and the analysis was performed well. There are a few points the authors might consider to improve the readability of the paper. 1) Some texts in the Results Section are repeating these in the Methods Section. The authors might consider removing them.

**Author Response:** Thank you for this comment – we have reviewed both sections and reduced redundant text as appropriate. In certain cases, text was maintained as we were mindful of readers who potentially review Results sections independently and would find it helpful to be briefly reminded of the related methods.

**Reviewer #2:** 2) The resolution of Figure 1 needs to improved. In particular, the texts are not readable.

**Author Response:** Figure 1 has been replaced for a high-resolution version, and we have uploaded this as a separate .tif file.

**Reviewer #2:** 3) A layout figure for the sensor and how the sensor was attached would be helpful.

**Author Response:** Figure 1 has been edited to include a schematic of the Earable device and labels of the specific sensors used in this pilot study.

**Reviewer #2:** 4) The type of intraclass correlation needs to be specified

**Author Response:** We computed the ICC1 Intraclass Correlation coefficient, where we calculated the between/between+within variance with respect to each subject (for each task). This has been clarified in the methods.

**Reviewer #3:** This is an interesting and well-conducted study. Can you explain the choice of the N=10 subjects and how that power was deemed adequate? Similarly, can you describe how the measures taken in healthy volunteers may differ in various patient populations that may benefit from the use of this device?

**Author Response:** Prior to this study, we did not perform any formal power calculations. The size of the pilot is determined by the feasibility of execution of a small study of heathy people. In this work we describe the development of features from the Earable device that allows powered hypothesis testing in a clinical or analytic validation study.

The use of the Earable device in disease populations (and larger control populations) is a future goal of this team. The data collection protocol and methods of analysis of this pilot study were designed to be utilized directly on data collected from patient populations, to limit low level technical issues that may arise when collecting actual clinical data—and we feel this study accomplished this goal. Given the study design and selection of PerfOs, we expect that the PerfO data distributions from patient populations would indeed differ from those computed from healthy volunteers, supporting the use of the Earable device for the evaluation of patient outcomes and diagnosis of relevant conditions to establish the magnitude of these differences (i.e. calculate effect sizes) in order to power clinical trials. Thus, the work presented here enables more accurate power calculations for future studies.
